# Lipidic Profile Changes in Exosomes and Microvesicles Derived From Plasma of Monoclonal Antibody-Treated Psoriatic Patients

**DOI:** 10.3389/fcell.2022.923769

**Published:** 2022-06-13

**Authors:** Giovanni Paolino, Sandra Buratta, Santo R. Mercuri, Roberto M. Pellegrino, Lorena Urbanelli, Carla Emiliani, Lucia Bertuccini, Francesca Iosi, Veronica Huber, Pina Brianti, Caterina Prezioso, Matteo R. Di Nicola, Cristina Federici, Luana Lugini

**Affiliations:** ^1^ Unit of Dermatology and Cosmetology, San Raffaele Hospital, Milan, Italy; ^2^ Department of Chemistry, Biology and Biotechnology, University of Perugia, Perugia, Italy; ^3^ Core Facilities, Microscopy Unit, Istituto Superiore di Sanità, Rome, Italy; ^4^ Unit of Immunotherapy of Human Tumors, National Institute of Tumors, Milan, Italy; ^5^ Department of Oncology and Molecular Medicine, Istituto Superiore di Sanità, Rome, Italy

**Keywords:** exosomes, inflammatory skin diseases, lipidic profile, microvescicles, monoclonal antibody treatment, psoriasis

## Abstract

Psoriasis is a chronic immune-mediated inflammatory skin disorder affecting children and adults. To date no approved biomarkers for diagnosis of this disease and follow up of patients have been translated into clinical practice. Recently, extracellular vesicles (EVs) secreted by all cells and present in almost all biological fluids are playing a crucial role in diagnosis and follow up of several diseases, including psoriasis. Since many psoriatic patients show altered plasma lipid profiles and since EVs have been involved in psoriasis pathogenesis, we studied the phospholipid profile of EVs, both microvesicles (MV) or exosomes (Exo), derived from plasma of psoriatic patients undergoing systemic biological treatment (secukinumab, ustekinumab, adalimumab), in comparison with EVs of untreated patients and healthy donors (HD). EVs were evaluated by immune electronmicroscopy for their morphology and by NanoSight for their amount and dimensions. EV phospholipid profiling was performed by High Resolution Liquid Chromatography-Mass Spectrometry and statistical Partial Least Squares Discriminant Analysis. Our results demonstrated that psoriatic patients showed a higher concentration of both MV and Exo in comparison to EVs from HD. The phospholipid profile of Exo from psoriatic patients showed increased levels of phosphatidylcholine (PC), phosphatidylethanolamine (PE), phosphatidylglycerol and lysoPC compared to Exo from HD. Sphingomyelin (SM) and phosphatidylinositol (PI) are the only phospholipid classes whose levels changed in MV. Moreover, the therapy with ustekinumab seemed to revert the PE and PC lipid composition of circulating Exo towards that of HD and it is the only one of the three biological drugs that did not alter SM expression in MV. Therefore, the determination of lipid alterations of circulating EVs could harbor useful information for the diagnosis and drug response in psoriatic patients.

## Introduction

Psoriasis is a common chronic immune-mediated inflammatory skin disorder, affecting the skin, nails and joints in children and adults, with an incidence in the general population ranging from 2.0% to 8.5% (Wu et al., 2016). The onset of a psoriatic lesion is a complex and multicellular process that involves keratinocytes, T cells, dendritic cells, macrophages, mast cells, endothelial cells and neutrophils. At the same time cytokines (i.e. TNFα, IL-17, IL-23) and growth factors initiate and sustain inflammation in this process (Rendon and Schäkel, 2019; Gisondi et al., 2020).

In psoriasis, biomarkers for disease prognosis and response to treatment are needed to help clinicians to improve patient management (Paolino et al., 2021a). Although many efforts have been made to identify psoriasis biomarkers (such as C-reactive protein, cytochrome-c, haptoglobin, platelet P-selectin, TNFα, IFN-γ, IL-6, IL-8, IL-12, IL-18 and IL-22), currently no biomarkers are used in clinical practice (Villanova et al., 2013; Paolino et al., 2021b). In addition, it is known that many psoriatic patients manifest altered serum lipid profiles with increased levels of high-density lipoproteins, apolipoprotein A1 and an augmented cardiovascular risk (Uyanik et al., 2002; Gisondi et al., 2020).

To date, there is no a curative therapy for psoriasis and the actual treatments for psoriasis can be divided in four main classes: topical treatments, phototherapy, systemic and biological therapies that improve clinical outcomes and quality of life (Feldman and Krueger, 2005; Kelly-Sell and Gudjonsson, 2017). Multiple classes of biologics are available: TNFα inhibitors, anti-p40 (IL-12/IL-23 antagonists), IL-17 and IL-17R inhibitors as well as the new anti p-19 inhibitors (selective for IL-23) (Kelly-Sell and Gudjonsson, 2017).

Extracellular vesicles (EVs) comprise microvesicles (MV), originating from the cell membrane with a diameter ranging from 150 to 1,000 nm, exosomes (Exo), deriving from the late endosomal multivesicular bodies with a size ranging from 30 to 150 nm, and apoptotic bodies, characterized by a dimension ranging from 1,000 to 5,000 nm (Caruso and Poon, 2018; van Niel et al., 2018). EVs are released by all cell types, in both normal and pathological conditions, and can be found in a wide range of bodily fluids (van Niel et al., 2018). They are composed of a lipid bilayer and carry different biological macromolecules (i.e., proteins, lipids, carbohydrates and nucleic acids). They play a major role in immune responses, cancer progression and metastasis, and inflammatory diseases (Stahl and Raposo, 2018). Recent studies indicate that EVs play key immunomodulatory roles in skin inflammatory disorders^,^ including psoriasis, atopic dermatitis, lichen planus, bullous pemphigoid, systemic lupus erythematosus, and wound healing (Shao et al., 2020). EVs can both stimulate and inhibit the innate and adaptive immune system (Théry et al., 2009; Federici et al., 2020; Zhou et al., 2020) and regarding their role in psoriasis, to date, only a few investigations have been carried out. A study revealed that EVs released from IFNα induced mast cells, could transfer cytoplasmic PLA_2_ to neighboring CD1a-expressing cells, which further led to the generation of neolipid antigens and subsequent recognition by CD1a-reactive T cells (Cheung et al., 2016). Moreover, EVs are critical mediators of keratinocyte-neutrophil crosstalk in the pathogenesis of psoriasis (Jiang et al., 2019; Nasiri et al., 2020). EVs derived from psoriatic keratinocytes also transferred miR-381-3p to CD4^+^ T cells, inducing Th1/Th17 polarization and promoting psoriasis development (Jiang et al., 2021).

Different studies highlighted the potential of EVs as diagnostic markers for psoriasis patients. The level of circulating EVs expressing IL-17A was higher in patients with moderate-to-severe psoriasis than in those with mild psoriasis (Jacquin-Porretaz et al., 2019). In comparison to healthy donors (HD), miRNAs of EVs from psoriasis patients showed an altered expression (Pasquali et al., 2020; Wang et al., 2020).

In immune-mediated diseases the number of circulating MV augments and this increase is particularly evident if vessels are also affected, possibly because a large part of circulating MV originates from platelets (Sellam et al., 2008; Baka et al., 2010; Krajewska-Włodarczyk et al., 2019). In psoriatic patients, the number of circulating endothelial and monocyte-derived MV increases (Takeshita et al., 2014) and no significant reduction of circulating endothelial-derived and platelet-derived MV was observed in patients successfully treated with anti-IL12/23 (Ho et al., 2016).

Lipid content of EVs has been poorly investigated with respect to protein and RNA content, even if it is well known that EV biogenesis requires the activation of specific lipid metabolizing pathways that, in turn, causes the partitioning of specific lipid classes in EVs (Ho et al., 2016; Sagini et al., 2018). The lipid cargo of EVs is also an important determinant for the uptake and cellular responses to target cells (Russell et al., 2019). EVs are enriched in cholesterol, sphingomyelin (SM), ether-linked phospholipids and lysoglycerophospholipids (lysoPL), compared to parental cells (Buratta et al., 2017; Buratta et al., 2021; Llorente et al., 2013; Phuyal et al., 2015). Another common characteristic is the high level of saturated phospholipids in EVs, compared to parental cells (Llorente et al., 2013; Lydic et al., 2015; Sagini et al., 2018). This feature is responsible for their high membrane stiffness, which is important to ensure stability and delay degradation in biological fluids (Record et al., 2018).

Targeted and untargeted LC-MS approaches quantifying bioactive lipid mediators in psoriasis patients and HD have depicted disease-specific phenotype profiles represented by polyunsaturated fatty acid (PUFA)-oxidized derivatives in both skin and blood (Zeng et al., 2017; Sorokin et al., 2018). Moreover, both pro- and anti-inflammatory eicosanoids were associated with joint disease scores in psoriatic arthritis patients (Coras et al., 2019).

Here, we investigated the lipid composition of two EV subtypes, MV and Exo, derived from plasma of psoriasis-affected patients (PSO) and HD. Furthermore, we studied the lipid profile of EVs isolated from plasma of PSO treated with secukinumab, ustekinumab and adalimumab monoclonal antibodies directed versus IL-17A, IL-12 and IL-23, and TNFα, respectively.

## Materials and Methods

### Human Subjects and Samples

All subjects with a diagnosis of psoriasis and with an age ≥18 (with or without joint involvement) were included in the present study. Patients with an age ≤17 years, patients with a diagnosis of a malignancy performed in the last 10 years, as well as patients with other inflammatory and/or autoimmune diseases were excluded from the present study. The present study was approved by the local ethics committee with the name of protocol: EXOPSO/2018.

The basic clinical-pathological variables (age, sex, type of psoriasis, BMI, comorbidities, therapy in progress, how long has psoriasis been) were reported in an anonymous file (Supplementary Table S1).

A total of 10 blood samples (each of 6 cc) from untreated PSO, 10 secukinumab-treated (SCK), 10 ustekinumab-treated (USTK) and 10 adalimumab-treated (ADM) patients, were analyzed and compared with 10 blood samples from HD. General median age of psoriatic patients was 50.5 (ranging between 22 and 70 years), with 27 males and 23 females. The main associated comorbidities were hypertension in 9 cases and hypercholesterolemia in 6 cases. General median age of healthy donors was 31 (ranging between 24 and 36 years).

### Isolation of Microvesicles and Exosomes From Plasma

Plasma was subjected to differential centrifugation as described before (Federici et al., 2020). Plasma, diluted 1:2 with PBS1X, was centrifuged for 30 min at 500 × g and 45 min at 12,000 × g to collect MV, followed by a washing step with PBS1X. Then plasma was filtered through a 0.22-µm filter (Sartorius, Germany), and ultracentrifuged for 2 h at 110,000 × g at 10°C to collect Exo, which were preserved at −80°C.

### NanoSight Analysis of EVs

Once isolated, the number and size of the isolated MV and Exo were evaluated by Nanoparticle Tracking Analysis (NTA; NanoSight Model NS300, Malvern Instruments, NanoSight Ltd., Salisbury, United States), using specific setting parameters for NTA capture (Paolino et al., 2021b). Briefly: camera type (sCMOS), Laser type Blue488, capture level 15, threshold 5, cursor gain (366) and capture duration (60 s). Data are expressed as mean ± SEM or mean ± SD, as indicated, using GraphPad Prism, and *p*-values of 0.05 or less were considered as significant. The statistical analysis was performed by paired and unpaired Student’s t-test, as indicated.

### Electron Microscopy Analysis of EVs

For electron microscopy, purified Exo and MV from 0.1 ml of plasma were processed for scanning electron microscopy (SEM) and transmission electron microscopy (TEM) as previously described (Shively and Miller, 2009; Paolino et al., 2021b).

For the immunoelectron microscopy, the samples on the grids were incubated with anti-CD81 monoclonal antibody (mAb B11, Santa Cruz Biotechnology, Heidelberg, Germany), and with 10 nm gold-conjugated goat anti-mouse immunoglobulin G (IgG) serum (Sigma-Aldrich, St. Louis, MO). Finally, nanovesicles were observed with a PHILIPS EM208S transmission electron microscope (FEI-ThermoFisher) (Federici et al., 2020).

### Lipid Analysis by High Resolution Liquid Chromatography-Mass Spectrometry (LC-MS)

Lipids were extracted from aliquots of Exo and MV (∼30 µg protein) as described (Matyash et al., 2008). Sample lipid profiles were analyzed by adapting the method previously described (Bird et al., 2011) to our LC/MS instrumentation (Agilent 1260 Infinity UHPLC system coupled to an Agilent 6530 Q-TOF). Peak detection, alignment, and lipid annotation were achieved using MassHunter Profinder (Agilent B.08.00) and a homemade adaptation of the LipidMaps database. Annotated lipids with quality identification score >90% were semi-quantified using the SPLASH I Lipidomix as standard reference. At the end of the workflow, the data matrix reporting data from 124 lipids was subjected to statistical analysis: sPLS-DA, Volcano Plot, Heatmap and Dendrogram were carried out on the MetaboAnalyst (5.0) web platform (Pang et al., 2021) Student’s test was applied to determine significant differences between two groups (*p* < 0.05) whereas ANOVA, followed by Tukey–Kramer post hoc test was applied to evaluate statistical differences between multiple treatment groups and controls.

## Results

### Plasma-Derived MV and Exo of Psoriatic Patients Versus HD

**FIGURE 1:**
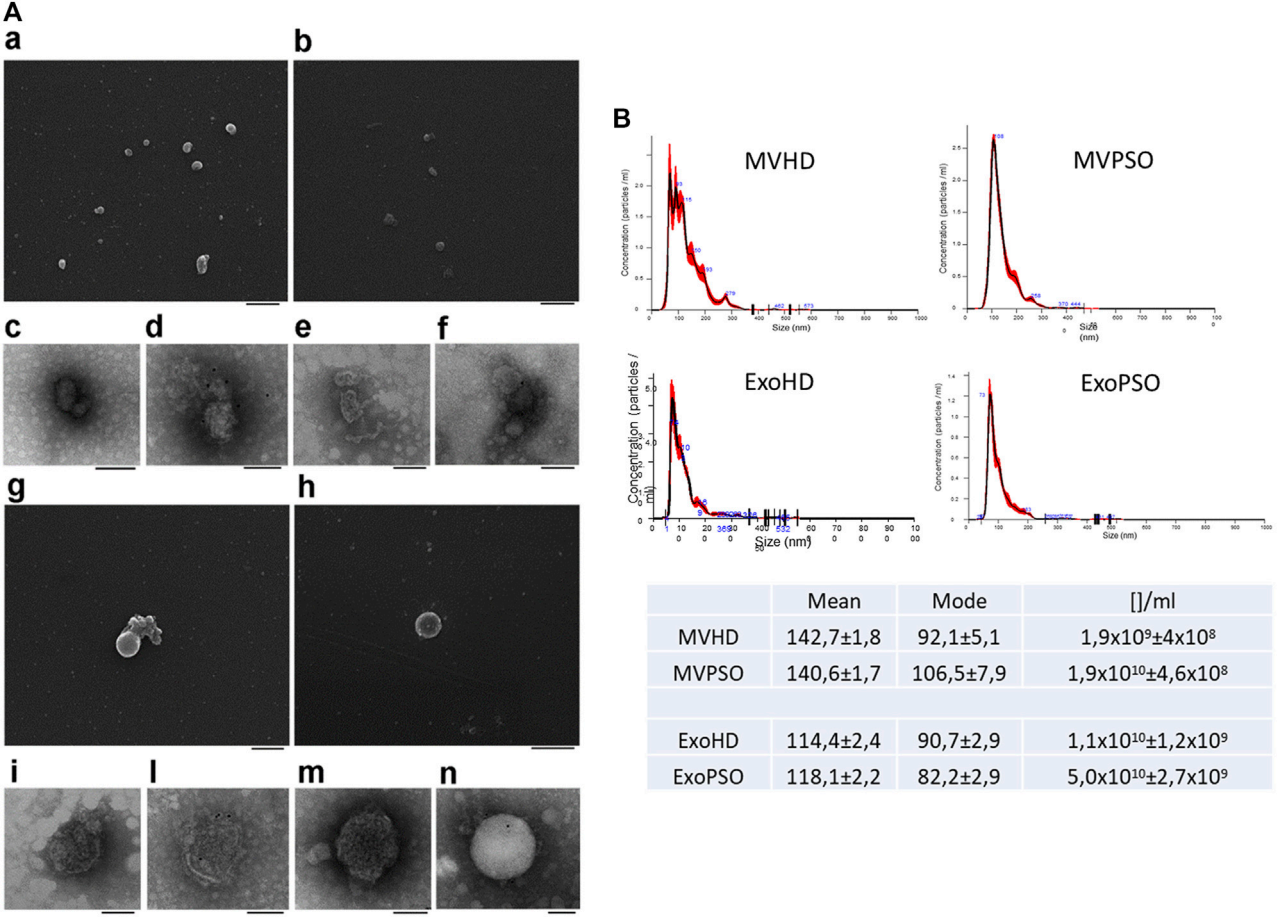
Morphological characterization of MV and Exo from plasma of HD and PSO. **(A)**. Scanning electron microscopy analysis of MVHD **(a)** and ExoHD **(b)**, Bars 500 nm. Transmission electron microscopy analysis of MVHD **(c)** and ExoHD **(e)**, Bars 200 nm. CD81-Immunoelectron microscopy of MVHD **(d)** and in ExoHD **(f)**, Bars 100 nm. Scanning electron microscopy analysis of MVPSO **(g)** and ExoPSO **(h)**, Bars 500 nm. Transmission electron microscopy analysis of MVPSO **(i)** and ExoPSO **(m)**, Bars 200 nm. CD81-Immunoelectron microscopy of MVPSO **(l)** and in ExoPSO **(n)**, Bars 100 nm. **(B)** Nanoparticle tracking analysis (NTA) analysis of MVHD and MVPSO (upper panels) and ExoHD and ExoPSO (lower panels). Representative spectra are shown. Mean, mode, and particles number/ml ([]/ml) are reported in the Table.

SEM analysis showed a major size of MV with respect to Exo and a round morphology for both MV and Exo (Figure 1A,a,b,g,h). TEM analysis confirmed the membranous nature of both EV sub-types (Figure 1A,c,e,i,m). Immunoelectron microscopy combined with positive/negative contrast method revealed the presence of CD81 EV marker (Figure 1A,d,l,f,n). NTA analysis confirmed bigger dimensions of MVHD and MVPSO (142.7 and 140.6 nm) as compared to ExoHD and ExoPSO (114.4 and 118.1 nm), respectively (Figure 1B), while no significative difference in size was observed between HD and PSO samples. Interestingly, psoriatic patients showed a higher concentration of both MV and Exo in comparison those from HD. In particular, the MV concentration in PSO was 10X higher with respect to HD, and Exo was 5X higher in PSO with respect to ExoHD (Table in Figure 1).

### Lipid Profile of Circulating EVs

#### Comparison of Lipid Profile of MV and Exo From Psoriatic Patient and HD

**FIGURE 2:**
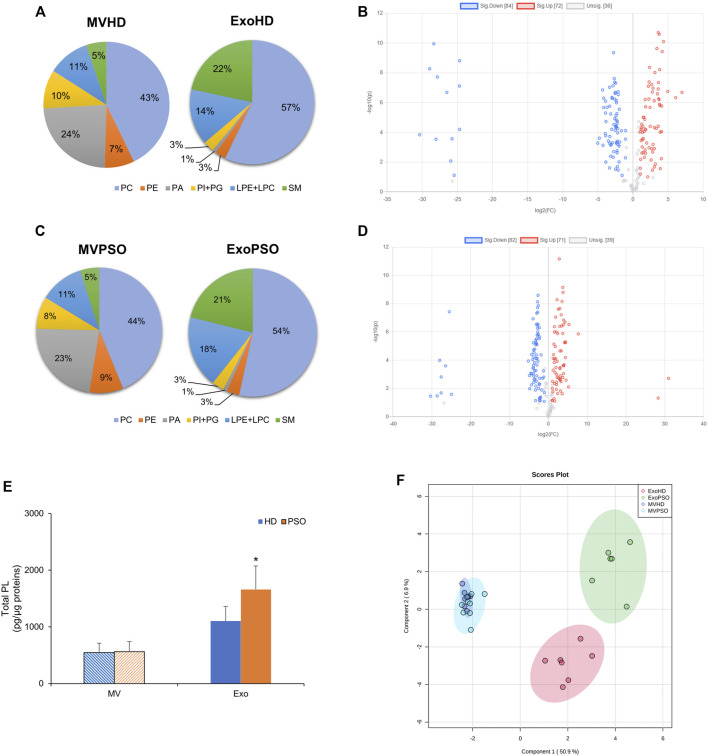
Statistical analysis of phospholipids detected in EVs from HD and PSO. **(A)** Pie charts of the percentage of each phospholipid class in MVHD and ExoHD. **(B)** Volcano plot of individual lipid species in MVHD and ExoHD. Lipids in blue and red were increased and decreased in MVHD vs. ExoHD, respectively (FC > 2 and *p*-value < 0.1). **(C)** Pie charts of the percentage of each phospholipid class in MVPSO and ExoPSO. **(D)** Volcano plot of individual lipid species in MVPSO and ExoPSO. Lipids in blue and red were increased and decreased in MVHD vs. ExoHD, respectively (FC > 2 and *p*-value < 0.1). **(E)** Amount of phospholipids (PL) relative to protein content. Data are reported as mean ± S.D. (*p < 0.05, PSO vs. HD). **(F)** PLS-DA of lipid species from HD and PSO, both for MV and Exo. 95% ellipses are constructed on each of the four groups. The dataset was normalised by median and scaled by Pareto algorithm.

We compared the lipid profile of plasma-derived EVs, both MV and Exo, from PSO and HD. The most abundant phospholipid classes in MVHD were phosphatidylcholine (PC) (45 + 11% of total PL), followed by phosphatidic acid (PA) (22 + 4.3% of total PL) and lysoPC (LPC) (10 + 2.1% of total PL). SM represented the 5.6 + 1.1% of total PL (Figure 2A). In Exo HD, PC represented the main PL class (58 + 8.4% of total PL), followed by SM (22 + 5.7% of total PL) and LPC (12 + 6.4% of total PL) (Figure 2A). No differences were observed in terms of percentage of PL classes among the HD and PSO samples (Figure 2A,C). However, the comparison between HD and PSO showed that ExoPSO presented higher PL/protein ratio, compared to ExoHD, whereas in MV no differences between the two experimental groups were measured (Figure 2E). In agreement with literature data, PS was not detected in our EVs samples (Sun et al., 2019; Jakubec et al., 2020).

Volcano plot analysis demonstrated the possibility to discriminate subtype on the basis of lipid composition. In HD, among the 192 lipid species detected, 43.75% were elevated and 37.5% reduced in MV as compared with Exo (fold change >2 and a corrected *p*-value < 0.1) (Figure 2B). Similarly, in PSO, among the 192 lipid species detected, 42.71% were elevated and 37.5% reduced in MV as compared with Exo (Figure 2D). These results indicate that the lipid content of MV is significantly different from that of Exo, both in normal and pathological samples.

Partial Least Squares Discriminant Analysis (PLS-DA) was applied to compare the lipid profile of the four experimental groups (MVHD, MVPSO, ExoHD, and ExoPSO) (Figure 2F). PLS-DA score plot indicated that component 1 separated MV from Exo, whereas component 2 separated ExoHD from ExoPSO, but not MVHD from MVPSO (Figure 2F). Thus, MVHD and MVPSO had a similar lipid asset, whereas ExoPSO showed a characteristic lipid composition that accounted for its clusterization in a separate group with respect to ExoHD.

#### Comparison of Lipid Profiles of EVs From Untreated and Antibody-Treated Psoriatic Patients, and HD

**FIGURE 3:**
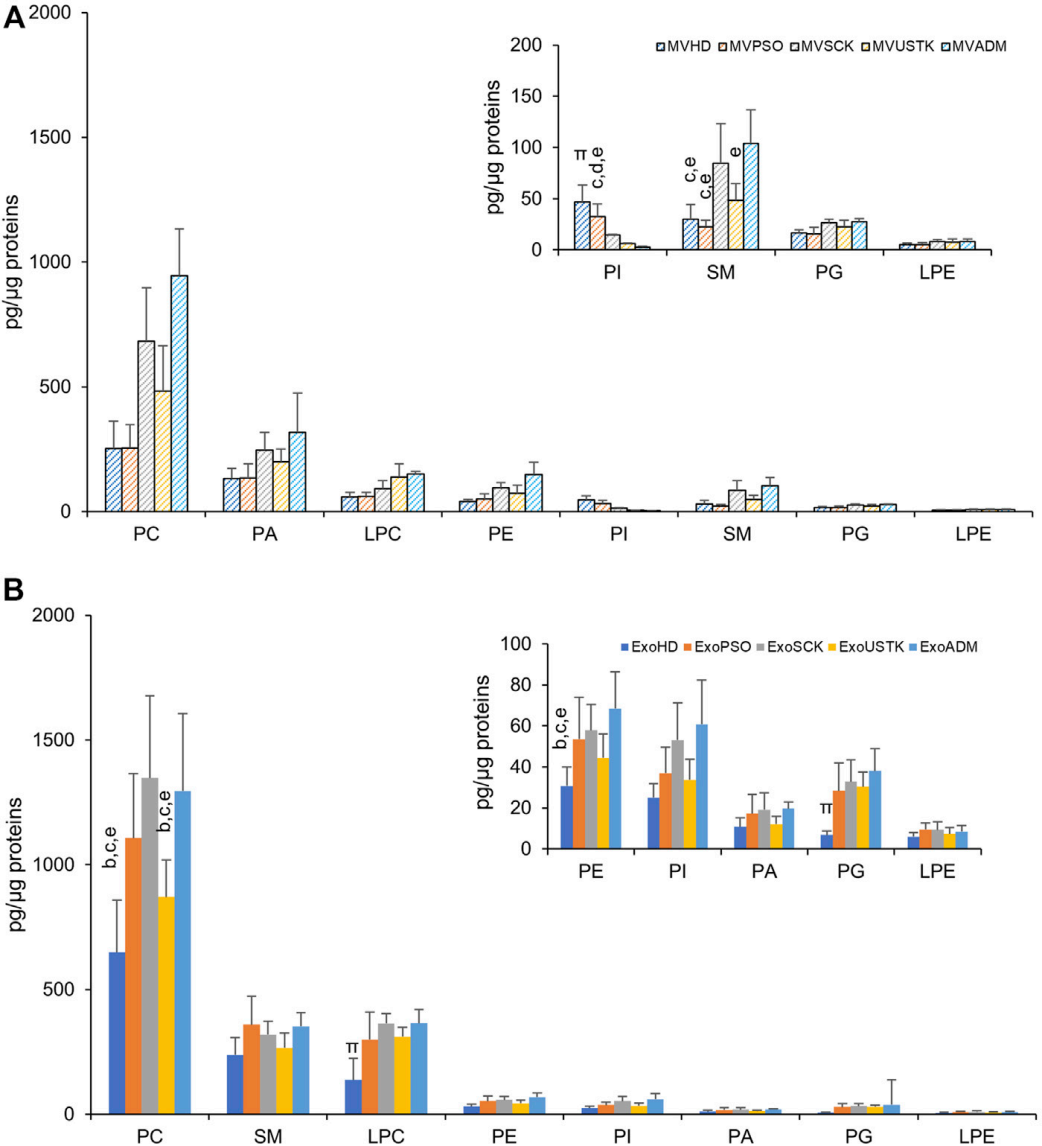
Phospholipid composition of EVs derived from plasma of HD, PSO and PSO treated with SCK, USTK and ADM. Lipid extracts from MV **(A)** and Exo **(B)** were analyzed by LC-MS. The amount of each PL class (sum of all detected species) is expressed as pg of lipids/µg of proteins. Data are reported as mean ± S.D. Statistically significant differences (*p* < 0.05) resulted by one-way ANOVA and Tukey’s post-hoc analysis; columns with different letters are significantly different (i.e. a vs. HD, b vs. PSO, c vs. SCK, d vs. USTK, e vs. ADM and π vs. all).

The amount of each PL class relative to protein content in MV (Figure 3A) and Exo (Figure 3B) from HD, PSO and from patients treated with SCK, USTK or ADM is reported. In MV, the amount of each PL class was comparable among the five experimental groups, except for SM and phosphatidylinositol (PI). Specifically, a higher content of SM was observed in MVSCK and MVADM, compared to the other groups. Lower levels of PI were observed in samples from drug-treated patients, compared with MVHD and MVPSO (Figure 3A). In Exo, we observed higher levels of PC, PE, phosphatidylglycerol (PG) and LPC in ExoPSO, ExoUSTK and ExoADM, compared to ExoHD. Notably, PC and PE levels of ExoUSTK were similar to ExoHD (Figure 3B).

**FIGURE 4:**
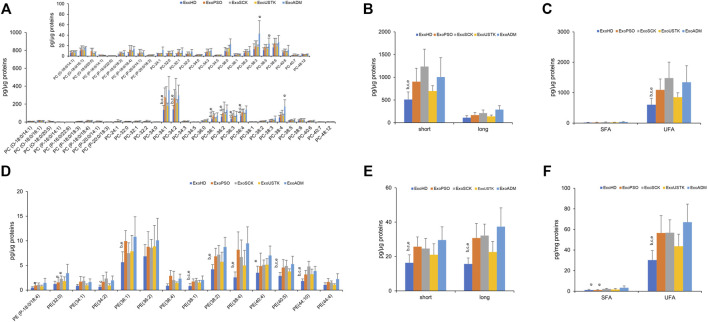
Molecular species of PC and PE detected in Exo from plasma of HD, PSO and PSO treated with SCK, USTK and ADM. Lipid extracts from Exo were analyzed by LC/MS–MS. Panels **(A)** and **(D)** show PC and PE detected species, respectively. Data are expressed as pg of lipid species/μg of proteins. The inserted panel expand the vertical axis to allow comparison of low abundance lipid subclasses. Panels **(B)** and **(E)** show fatty acid length in PC and PE, respectively. Data are reported as sum of species containing short (<36 carbon atoms) and long chains (>38 carbon atoms). Panels **(C)** and **(F)** show saturation level of PC and PE, respectively. Data are reported as sum of molecular species containing only saturated (SFA) and unsaturated (UFA) fatty acids. Data are reported as mean ± SD; *p* < 0.05 was considered statistically significant by one-way ANOVA and Tukey’s post-hoc analysis; columns with different letters are significantly different (i.e. a vs. HD, b vs. PSO, c vs. SCK, d vs. USTK, e vs. ADM and π vs. all).

We then reported changes in the molecular species belonging to PL classes whose levels were changed among the five experimental groups, in MV and Exo. In Exo, we evaluated changes in the PC and PE levels and calculated the length and saturation degree of acyl chains. Regarding PC, we detected three molecular species of PC-O (alkyl-phosphatidylcholine, PC with a fatty acid at the sn-1 position linked by a ether linkage while the fatty acid at the sn-2 position linked by an ester linkage to the glycerol moiety) (representing ∼3% of total PC), six molecular species of PC-P (phosphatidylcholine plasmalogens, PC with a fatty acid at the sn-1 position linked by a vinyl ether linkage while the fatty acid at the sn-2 position linked by an ester linkage to the glycerol moiety) (representing ∼2% of total PC) and 23 species of PC (diacyl-phosphatidylcholine) (Figure 4A). Statistical analysis revealed that levels of seven molecular species of PC were increased in ExoPSO, ExoSCK and ExoADM, compared with ExoHD and ExoUSTK. Noteworthy, these differences accounted for the greater content of PC observed in ExoPSO that was restored by USTK treatment to levels recapitulating ExoHD (Figure 3).

We also evaluated differences in the acyl chains length by calculating the content of PC species with short acyl chains whose sum composition was <36 carbons and long acyl chains whose sum composition was >38 carbons. As shown in Figure 4B, ExoPSO, ExoSCK and ExoADM presented greater levels of PC containing short acyl chains. ExoPSO, ExoSCK and ExoADM presented also higher levels of PC containing unsaturated fatty acids (UFA), compared to ExoHD and ExoUSTK (Figure 4C).

Regarding PE, levels of 10 out of 14 molecular species were changed among the five experimental groups (Figure 4D). The impact of these changes on PE properties is reported in Figure 4E,F. Higher levels of PE species containing short, long and unsaturated acyl chains were observed in ExoPSO, ExoSCK and ExoADM, compared to ExoHD and ExoUSTK (Figure 4E,F).

**FIGURE 5:**
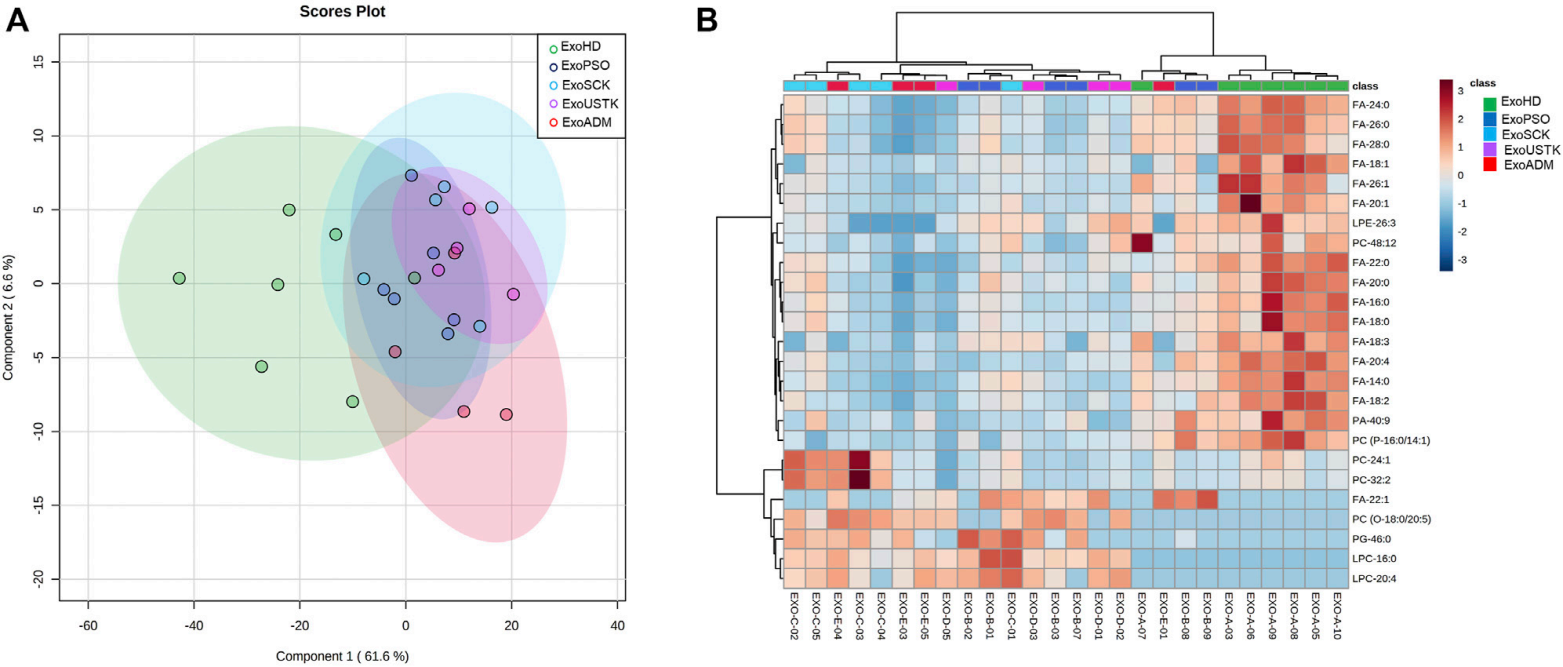
Chemometric and clustering analysis of Exo lipidome. **(A)** PLS-DA of five experimental groups based on the whole lipid data after median normalization and Pareto scaling. CI 95% ellipses are shown for the five different groups. **(B)** Heatmap of 25 most significant (ANOVA) lipids. Lipids and samples were both ordered using hierarchical clustering (Pearson distance). Autoscaled relative abundances of lipid species is represented by graduation of color from red (more abundant) to blue (less abundant).

PLS-DA was applied to analyze the five Exo groups based on the whole lipid data (Figure 5A). PLS-DA analysis displayed that the five lipidomic datasets clustered into two main groups. Exo from untreated and treated psoriatic patients were not clearly separated from each other but were well separated from ExoHD that clustered in a separate group (Figure 5A). The heatmap in Figure 5B was built using the 25 lipids that were most significant in the ANOVA test. The hierarchical analysis shows a clustering of the ExoHD against all other samples (Exo from untreated and treated patients). Among the 18 lipids overexpressed in ExoHD many were free fatty acids. The remaining seven lipids underexpressed in the ExoHD were predominantly glycerophospholipids.

**FIGURE 6:**
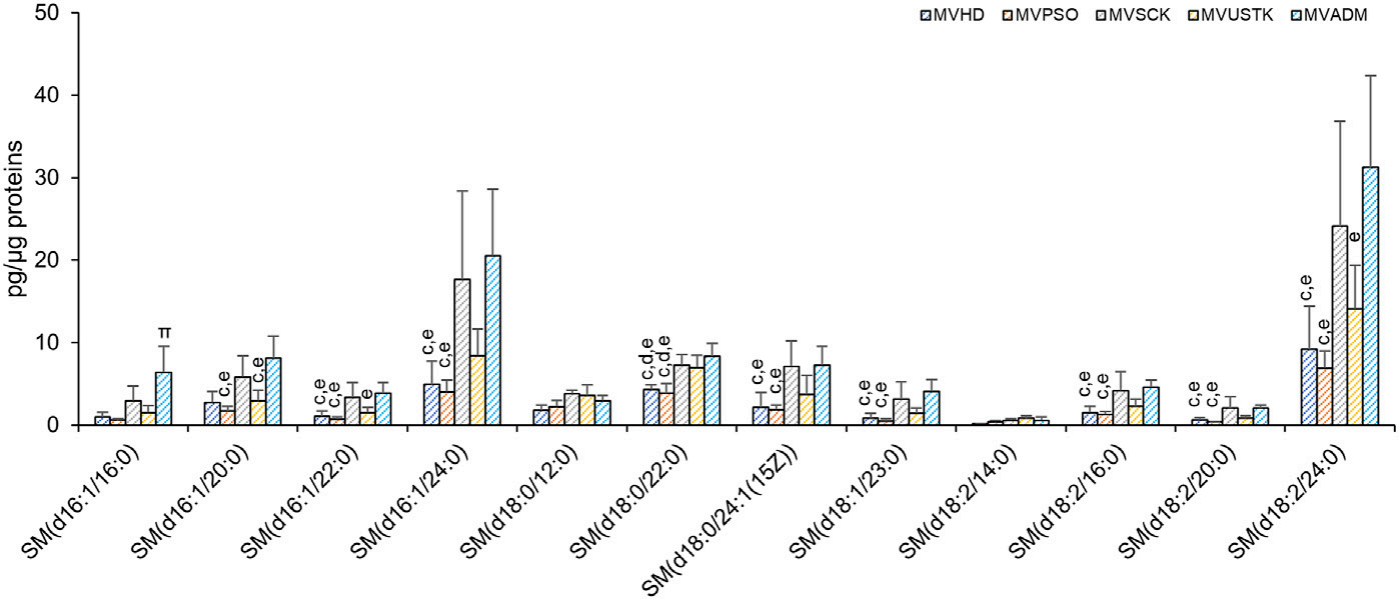
Molecular species of SM detected in MV isolated from plasma of HD, PSO and PSO treated with SCK, USTK and ADM. Lipid extracts were analysed by LC/MS-MS. The amounts of detected SMs were expressed as pg of lipid species/μg of proteins. Data are reported as mean ± SD; *p* < 0.05 was considered statistically significant by one-way ANOVA and Tukey’s post-hoc analysis; columns with different letters are significantly different (i.e. a vs. HD, b vs. PSO, c vs. SCK, d vs. USTK, e vs. ADM and π vs. all).

As reported in Figure 2, SM and PI are the only PL classes whose levels changed among the 5 MV groups. SM, and its derivative ceramide, are enriched in EV membrane and played a role in EVs biology (Verderio et al., 2018). In MV, we observed differences in the levels of 10 out of 12 molecular species of SM among the five experimental groups (Figure 6), which accounted for the higher content of SM in MVSCK and MVADM compared to the other groups (Figure 3A).

## Discussion

EVs are directly involved in the pathogenesis of inflammatory and autoimmune disorders (Wu et al., 2020), including psoriasis (Jiang et al., 2019; Shao et al., 2020). Despite this interesting evidence, few studies so far investigated the role of EVs in the pathogenesis of psoriasis (Jiang et al., 2019; Pasquali et al., 2020; Shao et al., 2020) and none of them focused on the lipid composition of EVs isolated from plasma of psoriasis-affected patients. It is known that there is a correlation between psoriasis and the increased amount of circulating lipids. Dysregulation of lipid metabolism is a pathogenetic feature of many diseases, including cardiovascular disease, hypertension, diabetes and Alzheimer’s diseases (Su et al., 2021) and in particular, psoriasis (Nowowiejska et al., 2021). Aberrations of lipid expression and metabolism, as well as lipid transporting proteins and receptors, are frequently present in psoriatic patients and these suffer more often from hyperlipidemia and are prone to develop metabolic syndrome, atherosclerosis, and thus cardiovascular disorders. (Nowowiejska et al., 2021).

In our study EVs, both MV and Exo, have been isolated from the plasma of HD and psoriasis patients, treated or not with three different biological drugs, i.e. monoclonal antibodies, and their lipid profile has been evaluated. We found that in treated and untreated psoriatic patients the amount of total circulating Exo and MV was significantly higher compared to HD and significative differences in size were appreciated comparing MV versus Exo.

MV and Exo showed a different lipid distribution. Both EV types presented similar levels of PC and LPC, but MV were enriched in PA whereas Exo were enriched in SM (Figure 2). Furthermore, Exo had a higher lipid/protein ratio, compared to MV. These results may be useful to discriminate among differently sized EV subpopulations, as the protein-based identification of EV subtypes may sometimes be controversial (Durcin et al., 2017), although without significant differences between PSO and HD samples.

The comparison between EVs from psoriatic patients and HD revealed that the most relevant differences were observed in Exo, while for MV the only difference regarded the level of SM. In line with this most of the studies focused on the correlation between EVs and psoriasis on exosomes (Jiang et al., 2019; Pasquali et al., 2020; Shao et al., 2020), which seem to be the main type of EVs involved in the pathogenesis of the disease. However, the global lipid profile analysis by PLS-DA indicates that MVHD and MVPSO had a similar lipid asset, whereas ExoPSO show a characteristic lipid composition that accounted for the clusterization of ExoPSO and ExoHD in two separate groups (Figure 2F). This result indicates that psoriatic patients and HD could be distinguished on the basis of lipid composition of plasma-derived Exo.

Furthermore, ExoPSO were characterized by higher levels of PC and PE, compared to ExoHD. Interestingly, in Exo from plasma of USTK-treated patients the levels of PC and PE were similar to the ones of ExoHD. The analysis of the molecular species of PC and PE revealed several acyl chain rearrangements that accounted for a greater level of short and unsaturated species in ExoPSO, compared with ExoHD. This result is important, taking into account the role of UFA as precursors of important lipid mediators. Indeed, with their phospholipid content, EVs represent a source of fatty acids that can be released by phospholipase A (Boilard, 2018). The signaling molecules derived from PUFA are called eicosanoids and are involved in important biological processes, including inflammation (Record et al., 2014).

Ustekinumab, the biological treatment that decreased the levels of PE and PC in ExoPSO to values comparable with ExoHD, is a monoclonal antibody that inhibits the p40 subunit common to IL-12 and IL-23, two cytokines involved in the pathogenesis of psoriasis. It has been shown that ustekinumab induces a reduction of IFNγ, TNFα, IL-8, IL-18, CD3^+^ T lymphocytes, and a reduction in the expression of many cytokine mRNAs involved in inflammation (Toichi et al., 2006). Most likely, ustekinumab by inhibiting the p40 subunit shared by IL-12, a helper T cell subtype 1 (TH1) inducer, and IL-23 (which maintains TH17 cell homeostasis) may attenuate inflammatory pathways and modulate the release and characteristics of circulating EVs (Poizeau et al., 2020). Further studies performed on larger groups of patients will be necessary to evaluate whether the same result can also be obtained with drugs that exclusively inhibit IL-23, which were not considered in the present study.

Interestingly, MV from plasma of patients treated with secukinumab and adalimumab were characterized by higher levels of SM, as compared to other MV samples (Figure 6). It is well known that SM and its metabolites, such as ceramide and sphingosine-1-phosphate (S1P), act as signaling molecules, controlling a vast number of cellular processes and sphingolipids playing important roles not only in EVs biogenesis but also in EV activity towards target cells (Verderio et al., 2018).

Moreover, in psoriatic patients we observed a decrease of total ceramide and an increase of S1P serum levels, reflecting their epidermal altered composition and metabolism. Patients with psoriatic arthritis have higher ceramide levels than psoriasis with skin involvement only (Myśliwiec et al., 2017).

Recently, sphingolipids have been identified among EV components as important diagnostic and prognostic biomarkers in cancer (Lydic et al., 2015; Skotland et al., 2017) and inflammatory disease (Moyano et al., 2016) and sphingolipid metabolites carried by EVs activate inflammation of macrophages (Kakazu et al., 2016). Therefore, differences in terms of SM may reflect an alteration of MV properties induced by modulation of inflammatory mediators.

Our study defines biochemical composition of circulating EVs in psoriasis and prompts the use of EV lipid cargo as possible biomarker source for assessing and following the status of psoriasis. Consistently, lipid content and, in particular, some lipid classes of circulating Exo (PC and PE) and MV (SM) can be potentially considered as determinants for the diagnosis of psoriasis, as well as means to follow the activity of the disease. Reversion of PC and PE levels in Exo from PSO after ustekinumab treatment to those measured in Exo from HD, suggests that Exo could be considered promising biomarker candidates of therapy outcome.

Future studies with a bigger sample size will better clarify the involvement of both Exo and MV in psoriasis. The monitoring of psoriasis could be improved through the lipid profile analysis of circulating EVs, correlating biochemical evidence with clinicopathological status.

## Data Availability

The original contributions presented in the study are included in the article/Supplementary Material, further inquiries can be directed to the corresponding author.
