# Supplementary material for: Lipidic Profile Changes in Exosomes and Microvesicles Derived From Plasma of Monoclonal Antibody-Treated Psoriatic Patients
Source: Front Cell Dev Biol. 2022 Jun 13;10:923769. doi: 10.3389/fcell.2022.923769 (PMC9234320; doi:10.3389/fcell.2022.923769)
Supplement: Supplementary file 1 [file Table1.DOCX]

|  | **Untreated Patients** | **Secukinumab** | **Ustekinumab** | **Adalimumab** | **Total** |
| --- | --- | --- | --- | --- | --- |
| **Male** | 6 | 6 | 9 | 6 | 27 |
| **Female** | 4 | 4 | 1 | 4 | 13 |
| **Mean age Tot.** | 54,6 | 49,8 | 44,1 | 49,5 | 49,5 |
| **Mean age M** | 52,83 | 47,83 | 41,1 | 47,83 | 46,7 |
| **Mean age F** | 57,25 | 52,75 | 71 | 52 | 55,31 |
| **Age Range Tot.** | 32 - 70 | 32 - 72 | 20 - 71 | 36 - 66 | 20 - 72 |
| **Age Range M** | 32 - 66 | 32 - 60 | 20 - 66 | 36 - 63 | 20 - 66 |
| **Age Range F** | 40 - 70 | 35 - 72 | 71 | 39 - 66 | 35 - 72 |
| **PSO vulgaris** | 8 | 9 | 10 | 10 | 37 |
| **PSO palmo-plantar** | 1 | 0 | 0 | 0 | 1 |
| **PSO palmo-plantar and arthropathic** | 1 | 0 | 0 | 0 | 1 |
| **PSO vulgaris and arthropathic** | 0 | 1 | 0 | 0 | 1 |
| **Time of PSO (mean, in years)** | 9,5 | 17,4 | 12,8 | 18,2 | 14,47 |
| **BMI (mean value)** | 27,14 | 27,66 | 25,87 | 27,17 | 26,89 |
| **Comorbidities (patients number)** | 3* | 6** | 4*** | 4**** | 17***** |

PSO means psoriasis; BMI means body mass index

Comorbidities: * 1 epilepsy; 1 hypertension; 1 hypertension/diabetes/hypercholesterolemia ** 2 hypercholesterolemia; 2 hypertension; 1 hypertension/hypercholesterolemia; 1 thyroiditis ***1 hypertension/glaucoma; 1 hypertension/thyroiditis; 1 diabetes/hypercholesterolemia/previous heart attack; 1 hypercholesterolemia ****1 diabetes; 1 hypertension/hyperuricemia; 1 hypertension/thyroiditis; 1 thyroid nodules ***** 1 diabetes; 1 diabetes/hypercholesterolemia/previous heart attack; 1 epilepsy; 3 hypercholesterolemia; 3 hypertension; 1 hypertension/diabete/hypercholesterolemia; 1 hypertension/glaucoma;1 hypertension/hypercholesterolemia; 1 hypertension/hyperuricemia; 2 hypertension/thyroiditis;1 thyroid nodules; 1 thyroiditis
